# Cross-Cultural Adaptation and Validation of the Romanian Musculoskeletal Tumor Society Scoring System for Patients with Extremity Bone Sarcomas

**DOI:** 10.3390/medicina60050778

**Published:** 2024-05-08

**Authors:** Adyb-Adrian Khal, Dragos Apostu, Rodica Cosnarovici, Sébastien Pesenti, Jean-Luc Jouve, Razvan-Catalin Mihu

**Affiliations:** 1 Department of Orthopaedics and Traumatology, Iuliu Hatieganu University of Medicine and Pharmacy, 400000 Cluj-Napoca, Romania; apostudragos@yahoo.com; 2 Department of Paediatric Orthopaedics, AP-HM Timone Enfants, 13005 Marseille, France; sebastien.pesenti@ap-hm.fr (S.P.); jean-luc.jouve@ap-hm.fr (J.-L.J.); 3 Department of Paediatric Oncology, “Ion Chiricuta” Oncology Institute, 400015 Cluj-Napoca, Romania; rodicacosnarovici@yahoo.com; 4 Department of Orthopaedics and Traumatology, Regional University Hospital, 410169 Oradea, Romania; razvan_mihu@yahoo.com

**Keywords:** bone sarcoma, bone reconstruction, functional outcome, MSTS, Romania

## Abstract

*Background and Objectives:* Primary malignant bone tumors are rare lesions, and their complex treatment can lead to functional impairment. It is important to have a postoperative assessment tool for patients’ functional outcomes to be evaluated and to consequently adapt future treatments in the pursuit of a continuous improvement of their quality of life. The Musculoskeletal Tumor Society Score (MSTS) is a validated specific system score that is used frequently in the follow-up of these patients. We found no information about a valid translated Romanian version of this score neither for the upper limb nor for the lower limb. We proposed in this study to translate the original version of the MSTS Score into Romanian and to perform validation analysis of the Romanian-language MSTS Score. *Materials and Methods:* We selected 48 patients who underwent limb-salvage surgery after resection of bone sarcomas. Patients were interrogated twice according to the translated Romanian version of the MSTS Score during their follow-up. The translation was performed according to the recommended guidelines. A total number of 96 questionnaires were valid for statistical analysis. *Results:* Internal consistency and reliability were good for both sets of questionnaires’ analytic measurements, with Cronbach’s alpha values of 0.848 (test) and 0.802 (retest). The test-retest evaluation proved to be statistically strong for reproducibility and validity with Spearman’s rho = 0.9 (*p* < 0.01, 95% CI). *Conclusions:* This study permitted the translation of this score and the validation of psychometric data. Our results showed that the Romanian version of the MSTS is a reliable means of assessment of the functional outcome of patients who received limb-salvage surgery for the upper and lower extremities.

## 1. Introduction

Primary malignant bone tumors are rare and they represent approximately 1% of all existing malignant lesions [1,2,3]. The global incidence is about 5% per 100,000 inhabitants per year [1,2,3]. It is considered that bone sarcomas have an incidence of one new case per 100,000 inhabitants per year [1,2,3]. Among them, the most common are osteosarcomas, chondrosarcomas, and Ewing sarcomas.

In the late 1970s, with the thorough histopathological studies conducted by Mario Campanacci [4], the introduction of different grading systems according to the degrees of malignancy of these lesions by Broder [5], the introduction of adjuvant and neo-adjuvant chemotherapy by Rosen [6] and Huvos [7], and musculoskeletal oncology became common language with the efforts of Enneking [8] to describe the first staging system. Since then, research has permitted the conceptualization of precise chemotherapy and radiation therapy protocols together with time-framed surgical intervention to allow treatment of these lesions [9,10].

The local treatment of these malignancies consisted, previously, of first-line radical surgeries such as amputation or disarticulation with a strong psychological impact both for the patient and for society [2,11,12]. Nevertheless, with the large-scale evolution of accurate diagnostic imaging tools, precise chemotherapy and radiotherapy protocols, and especially limb-salvage surgery, the quality of life of patients affected by musculoskeletal tumors has increased significantly [1,2,11]. Various alternative reconstruction techniques have led to a change in the concept of treating musculoskeletal oncological patients; now, the purpose is not only to save lives but also to improve the functional outcome of these patients.

From this perspective, it was important to have a postoperative assessment tool for patients to determine functional outcomes. The Musculoskeletal Tumor Society Score (MSTS) was developed in 1985 and revised in 1993 [13]. It has been accepted as an international score by the Musculoskeletal Tumor Society (MSTS) and by the International Society for Limb Salvage (ISOLS) [13]. Since then, this system has been used in numerous studies to assess functional outcomes, making it the most widely used functional evaluation tool [13].

The original MSTS Score was developed in the English language as were most questionnaires. However, in multicenter international studies and registries, it is necessary to provide questionnaires and tools for patients in the language of each country included. In addition, within each country, it is relevant to assess the MSTS score even in the presence of significant cultural and ethnic diversity in the population due to the presence of immigrant populations, especially when their exclusion could lead to a systematic bias in studies of healthcare utilization or quality of life. Over the last decades, the MSTS Score has been secondarily translated into several languages such as French, Greek, Danish, Brazilian, Turkish, Japanese, and Chinese in order to better record information on the functional follow-up of these patients across different countries [14,15,16,17,18,19,20,21]. Due to the available guidelines for cross-cultural agreement, reproducibility, and validation of different scores [22,23], translation into other languages in non-English-speaking countries has become possible with global adaptation and valid context. With a population of 18 million inhabitants in Romania, plus approximately six million living abroad, a Romanian version of the MSTS score has not been validated until now.

Therefore, the aim of this study was to implement this score in our language and to perform validation analysis of the Romanian translation of the MSTS Score in patients who underwent limb-salvage surgery after resection of bone sarcomas.

## 2. Materials and Methods

Patients who underwent surgical treatment for primary malignant bone tumors in a single referral center between 2010 and 2021 were retrospectively evaluated. The inclusion criteria for enrolling patients were as follows: positive histopathological diagnosis of bone primary malignant tumors of the upper or lower limb according to WHO 2020 classification [24], age range between 0–70 years, localized lesion with no metastasis at diagnosis, no confirmed local recurrence nor distant metastasis during the follow-up, at least 12 months of follow-up after reconstructive limb-salvage surgery and patients being native Romanian speakers. A total of 48 patients met all the criteria and agreed to participate. The clinical and demographic data are detailed (Table 1). All patients (including the parents of a minor patient) gave written consent to participate in the study. The study was conducted according to the guidelines of the Declaration of Helsinki and it was approved by the local Ethics Committee of the local institution (127–19 March 2019).

The use of radiotherapy and chemotherapy was decided at the discretion of a multidisciplinary team (orthopedic surgeon, radiotherapist, and medical oncologist). In the case of osteosarcoma, patients received chemotherapy according to the EURAMOS protocol [9] and, in the case of Ewing Sarcoma, patients received Euro Ewing chemotherapy protocols according to the time frame [10].

All resected specimens were analyzed for surgical margins according to Enneking criteria [8].

Primary malignant bone tumors that were identified in our patients were: Ewing Sarcoma (20.8%, 10/48) with molecular biology confirmation of EWSR1 chromosomal translocation; conventional chondrosarcoma (8.3%, 4/48), and osteosarcoma (70.9%, 34/48) with the following histotypes: conventional osteoblastic (*n* = 28), chondroblastic (*n* = 2), parosteal osteosarcoma (*n* = 2), teleangiectatic (*n* = 1), and small-cell osteosarcoma (*n* = 1).

In the follow-up, patients were evaluated every 3 months during the first two years, every 6 months up to the 5th year after surgery, and once a year until ten years after surgery by a team of orthopedic surgeons. Clinical and radiological examination included the assessment of the stability of the reconstruction and the range of motion. All complications were recorded and described according to the Henderson classification [26]. Patients were functionally evaluated with the Musculoskeletal Tumor Society (MSTS) Score.

The MSTS scoring system is made up of a total of six parameters [13]. Three parameters are subjective to the patients (pain, functional activities, and emotional acceptance) and the other three parameters are specific to the patient’s tumoral site in the upper or lower limb [13]. Upper limb parameters consist of hand positioning, manual dexterity, and lifting abilities while lower limb parameters consist of the use of external supports, walking ability, and gait [13]. The clinician assigns each parameter a point from 0 to 5, with the total score being between 0 to 30 [13]. A higher MSTS value corresponds to better limb function: poor (<15 or <50%), fair (15–17 or 50–59%), moderate (18–20 or 60–69%), good (21–22 or 70–74%), and excellent (23–30 or 75–100%) [13].

The Romanian translation was conducted according to Beaton’s [22] and Guillemin’s [23] criteria by a team of 3 native Romanian orthopedic surgeons (AAK, DA, RCM) who were proficient in English. Professional medical translators were not necessary because each sentence was relatively simple. All independent translations were compared and a common scoring document was approved by each member of the team. Therefore, the Romanian-translated version of the MSTS Score was re-translated into English to check for major differences between the original version and ours (Table 2 and Table 3). From the viewpoint of cross-cultural adaptation, no modification was necessary to our version as each item description in the original version fits well with the modern Romanian lifestyle. Subsequently, 2 members of the team (RCM, RC) conducted separate interviews with each patient on different occasions to analyze the validity and reproducibility of the final version. The second completion of the same questionnaire was achieved during the follow-up by a different interviewer (AAK) so all patients were presented with the questionnaire twice, with at least 2 weeks between the test and retest. All results were gathered in a common document with no further discrepancies being found.

A descriptive study is presented for the patient’s demographic information and data are presented in total frequencies and percentages. The Romanian version of the MSTS score was statistically analyzed for internal consistency, reliability, reproducibility, and validity. Internal consistency and reliability were determined using Cronbach’s alpha and the intraclass correlation coefficient (ICC). Cronbach’s alpha index ranges from 0 to 1 and a 0.7 value was used as a benchmark to determine if the internal consistency was satisfactory. All patients repeated the test for the test-retest assessment. Reproducibility and validity were evaluated using Spearman’s correlation coefficient calculation (Spearman’s Rho index). A benchmark of 0.7 < rho < 1 was used to interpret Spearman’s correlation coefficient as satisfactory.

## 3. Results

**Figure 1:**
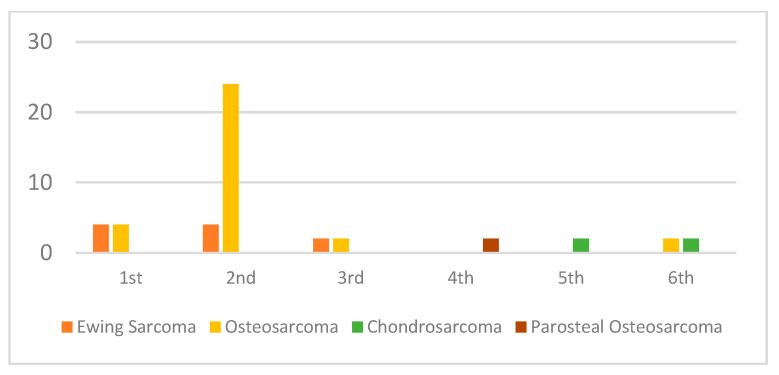
Group age variations by decade of life and histopathology of bone lesion.

The male to female ratio was 28:20 (1.4:1) and the mean age at the time of surgery was 19.8 years (range, 7–53). Demographic data are presented in Table 1 and Figure 1.

Initial symptoms were a swollen mass in 24 patients (50%, 24/48), pain in 21 patients (43.75%, 21/48), and pathological fractures in two patients (4.15%, 2/48). The average duration of symptoms prior to diagnosis was 3.8 months (range, 2–7). One patient (2.1%, 1/48) was asymptomatic and the lesion was discovered incidentally during post-traumatic radiography.

All patients received chemotherapy, except those (n = 6, 12.5%) who had conventional chondrosarcoma or low-grade osteosarcoma, such as parosteal osteosarcoma.

All patients had negative resection margins. Radiation therapy was not necessary either pre-operatively or post-operatively.

Limb-salvage surgeries were performed on all patients. Massive endoprosthesis (simple and expandable) was the main means of limb salvage (n = 28). Biological reconstruction was performed on 18 patients and shoulder arthrodesis on two patients.

Complications occurred in 26 patients (54.1%) (Table 4). Out of these, 14 patients had an endoprosthetic reconstruction and 12 patients had a biological reconstruction. Twelve patients (25%) had two or more types of complications during follow-up. Additional surgeries were required in all 26 patients, most of them for mechanical complications. Pseudarthrosis was the most frequent complication (30.8%) and occurred in eight pediatric patients who underwent biological reconstruction with either an epiphyseal vascularized transfer or a vascularized fibula. These patients underwent revision surgery with autologous grafting. Fractures of the biological graft (23.1%) were subjected to surgical reintervention which consisted of autografting and bone fixation. Deep infection (23.1%) occurred in patients with biological reconstruction or with endoprosthesis. They all required surgical debridement and antibiotic therapy based on an antibacterial susceptibility test. Partial or total prosthesis revision was performed in case of prosthesis loosening (7.7%).

A total number of 96 questionnaires (test-retest) were valid for statistical analysis. No ethical considerations or challenges were encountered during the translation process. No patient declined to complete the test. There were no difficulties in completing the questionnaires. All analyzed questionnaires were fully completed.

The mean MSTS Score was 23.33 (range, 15–29) for the first round of interrogations, with separated means of the MSTS score being 22 (range, 15–29) for the patients with upper limb sarcoma (Table 5) and 23.77 (range, 19–28) for the patients with lower limb sarcoma. The mean MSTS Score was 23.91 for the retests. Patients with upper limb sarcoma had an average of 23.1 (range, 20–29) on retests (Table 6), while patients with lower limb sarcoma had an average of 24.1 (range, 19–29).

Internal consistency and reliability were good for the analytic measurements of both sets of questionnaires. For the first test, Cronbach’s alpha was 0.848 and the intraclass correlation coefficient (ICC) was 0.482 for single measures (95% CI, 0.358–0.616) and 0.848 for average measures (95% CI, 0.770–0.906). For the retest, Cronbach’s alpha was 0.802 and the intraclass correlation coefficient (ICC) was 0.403 for single measures (95% CI, 0.280–0.544) and 0.802 for average measures (95% CI, 0.700–0.877).

The test-retest evaluation proved to be statistically strong for reproducibility and validity. Spearman’s rho has an index value of rho = 0.9 (*p* < 0.01, 95% CI); thus, the tests and retests have a strong positive monotonic correlation. No floor or ceiling effects were encountered during the first or second completions of the questionnaires.

## 4. Discussion

Primary malignant bone tumors of the upper limb or lower limb have a relevant impact on the quality of life of patients, due to a significant impairment in domains that include daily activities, physical function, relational function, pain, psychological health, and social roles. In these patients, apart from neo-adjuvant or adjuvant therapies such as chemotherapy or radiation therapy, complex orthopaedical surgical interventions may bring additional risk factors for functional disabilities. Even if recent reconstruction techniques have led to a change in the concept of treating musculoskeletal oncological patients and the continuous improvement of functional outcomes has become a fundamental purpose for orthopedic surgeons, postoperative complications still occur. Each complication with consequent additional surgeries may result in a lower functional outcome. In the follow-up, the most important factor that influences the MSTS score is the occurrence of complications and these can cause changes in both the patient’s subjective indices and the objective indices [13,27,28,29].

The literature concerning clinical and mechanical outcomes in bone sarcoma healthcare is characterized by outcome measures of function. Moreover, over the past decades, there has been an increasing amount of literature reporting an improvement in the quality of life outcomes in these patients [11,12,27,28,29,30]. This way, the MSTS score has played an important role in the follow-up process of patients who underwent limb-salvage surgery for bone sarcomas [31,32,33,34,35].

The implementation of several translated versions of the MSTS score in different languages is helping clinicians in assessing patients’ outcomes. The Romanian translation of the MSTS score is valid and has been tested statistically for consistency, reliability, and reproducibility. Our results are comparable to the other published literature articles regarding cross-cultural adaptations of the MSTS score: Brazilian (Cronbach’s alpha of 0.84 [18]), Chinese (Cronbach’s alpha of 0.86 [21]), Japanese (Cronbach’s alpha of 0.87 [19,20]), Danish (Cronbach’s alpha of 0.85 [16]), Greek (Cronbach’s alpha of 0.76 [15]), French (Cronbach’s alpha of 0.83 [14]), and Turkish (Cronbach’s alpha of 0.97 [17]).

We reported two coefficients with their respective 95% confidence intervals for the intraclass correlation coefficient (ICC). Single measures of the ICC showed reliability ratings for one, typical, single rate, while average measures of the ICC test and retest showed reliability of different rates averaged together. Single measures of ICC scored under 0.5 do not reveal a true lack of reliability but a certain degree of heterogeneity of the studied group. However, this was expected due to the complexity and differences between the studied patients in terms of clinical examination, diagnosis, surgical approach, and anatomopathological results. Average measures of the ICC scored excellent in terms of reliability for averaged rates, thus demonstrating that the Romanian adaptation of the questionnaires and MSTS score is successful and fit for clinical use.

Thorough clinical examination and investigation of the patients during the follow-up measured functionality after limb-salvage reconstruction. We performed an in-depth analysis of the correlation between the personal MSTS score and the clinical status of every patient. According to the results, the translated MSTS individual scores are valid and in strong correlation with the patient’s functional status. Furthermore, all separated items were proven for validity. Subjective items such as pain and emotional acceptance showed strong validity but scored relatively lower than the objective items such as walking or lifting ability due to personal bias related to the patients. No statistical difference was present between the validity parameters regarding age or gender.

The Romanian MSTS score validation study reinforces the local rise of general awareness and knowledge regarding musculoskeletal sarcoma. Along with the development of surgical techniques and post-operative care, there is a rising need for a universal evaluation scale. The scheme of this instrument that measures physical functionality as well as the patient’s quality of life makes the usage of this scale desired. Throughout our study, we obtained consistent, reliable, and valid results showing that limb-salvage surgery provides very good outcomes for patients with upper- or lower-extremity musculoskeletal sarcoma.

This is the first time the MSTS scale was successfully translated and adapted into Romanian. Our cross-cultural adaption of the MSTS scale included both the upper and lower limb versions, making the test translation representative as a future clinical evaluation tool. The high values of statistical indices show that there are no cultural or linguistic differences between the original English version and the translated Romanian version. Moreover, in this relatively small series of patients, we did not find any unexpected findings or patterns concerning the follow-up of these patients. Our complications rate (54% vs. 40–80%) and functional outcomes are comparable to other studies in the literature, emphasizing the universal standardized practice of limb-salvage surgery in our country [11,14,15,26].

This study has several limitations to be considered. First, the study was retrospective and, therefore, it has an inferior level of evidence. We strongly agree that all malignant bone lesions need an appropriate approach concerning their histopathological features, but we did not perform a detailed histopathological analysis of our cohort as the purpose was to focus on the functional outcome. However, important oncological details are stated to offer a clear summary of all the retrospective data of patients that were included. Patients were recruited in a single center by convenience sampling and are thus not representative of the general population and prone to selection bias and inherent limitations. However, in our country, five national oncological institutes exist, each of them being referenced by almost 30% of the population. It may not be possible to judge the true incidence of complications due to the limited sample size; a larger sample size is therefore recommended to clarify this result in future studies. Second, we did not investigate the relationship between the MSTS Score and the type of surgery, nor the relationship between the anatomical compartments and the natural barriers involved in neoplastic growth. The technique of reconstruction was not randomized, and the preference of the surgeon may have contributed to a selection bias. Pediatric patients completed the questionnaires together with their parents, thus the appearance of subjectivity being probable; patients’ age repartition was the same in our study as in other MSTS cross-cultural validation studies.

However, primary bone tumors are rare, and, to the best of our knowledge, we report the first cross-cultural analysis of the MSTS Score in the Romanian population. This information can help surgeons counsel patients in terms of functional outcomes after limb-salvage reconstruction for patients with bone sarcoma. This translated questionnaire may be used in follow-up consultations to standardize the functional results of these complex surgeries. This might increase awareness and strengthen the long-term purposes in treating bone sarcoma patients, where the aim is not only to save lives but also to improve the functional outcomes of these patients. The patient–surgeon- or patient–medical oncologist-specific relationship may be improved on the basis of confidence towards high-quality mechanical outcomes. Our study does not advocate specific training programs during long-term rehabilitation, but based on the knowledge of what is possible in bone sarcoma survivors, the results are satisfactory.

## 5. Conclusions

In conclusion, we have translated the MSTS Score into Romanian and validated the questionnaire. Our results showed that the Romanian version of the MSTS is a reliable means of assessment of the functional outcomes of patients who received limb-salvage surgery on their upper and lower extremities. In this population, it is a valid instrument that can be utilized for the follow-up of patients with primary malignant bone tumors.

## Figures and Tables

**Table 1:** Demographics and clinical data for patients included in the study.

**Characteristics**	**Value**
**Mean age, years (range)**	19.8 (7–53)
**Gender: Female, n (%)** **Male, n (%)**	20 (41.7) 28 (58.3)
**Histology, n, (%)**	Chondrosarcoma: 4 (8.3) Ewing Sarcoma: 10 (20.8) Osteosarcoma: 32 (66.7) Parosteal Osteosarcoma: 2 (4.2)
**Stage Enneking at diagnosis, n (%)**	I B: 2 (4.2) II B: 42 (87.5) III: 4 (8.3)
**Localization:** **Upper limb** **Proximal Humerus, n (%)** **Metacarpus, n (%)** **Lower limb** **Proximal Femur, n (%)** **Femur Diaphysis, n (%)** **Distal Femur, n (%)** **Proximal Tibia, n (%)** **Distal Tibia, n (%)** **Distal Fibula, n (%)**	10 (20.8) 2 (4.2) 2 (4.2) 4 (8.3) 22 (45.8) 4 (8.3) 2 (4.2) 2 (4.2)
**Neo-adjuvant Radiotherapy, n (%)** **Yes:** **No:**	0 (0) 48 (100)
**Chemotherapy, n (%)** **Yes:** **No:**	42 (87.5) 6 (12.5)
**Type of limb salvage surgery, n (%)** **Allograft** **Shoulder Arthrodesis** **Capanna technique** [25] **Epiphyseal transfer** **Expandable prosthesis** **Massive endoprosthesis** **Non-vascularized autograft** **Vascularized fibula**	2 (4.2) 2 (4.2) 2 (4.2) 6 (12.5) 6 (12.5) 22 (45.8) 2 (4.2) 6 (12.5)
**Complications, n (%)** **Yes:** **No:**	26 (54.2) 22 (45.8)
**Reintervention in patients with complications, n (%)** **Yes:** **No:**	26 (100) 0 (0)
**Mean follow-up time, months (range)**	54 (12–146)

**Table 2:** English translation of the Romanian-translated MSTS Score for the upper limb.

*Description*	*Data*	*Score*
**Hand position**		
Unlimited	180° elevation	5
Intermediary		4
Not above the shouler/No pronosupination	90° elevation	3
Intermediary		2
Not above the hip	30° elevation	1
Without	0	0
**Manual dexterity**		
Unlimited	Normal dexterity and sensibility	5
Intermediary		4
Loss of fine movements	Cannot button/Slight loss of sensibility	3
Intermediary		2
Cannot clip	Major loss of sensibility	1
Cannot catch	Anesthesia	0
**Lift ability**		
Normal	Normal	5
Intermediary	Slight limitation	4
Limited	Light objects lifting	3
Intermediary	Only gravity	2
Only with help	Impossible against gravity	1
Impossible	Cannot move	0
**Pain**		
No pain	No medication	5
Intermediary		4
Moderate/No invalidity	Non-narcotic analgesia	3
Intermediary		2
Moderate/Intermittent invalidity	Intermittent narcotics	1
Severe/Continuous invalidity	Continuous narcotics	0
**Function**		
No restriction	No invalidity	5
Intermediary		4
Recreational restriction	Minor invalidity	3
Intermediary		2
Partial occupational invalidity	Major invalidity	1
Total occupational invalidity	Complete invalidity	0
**Emotional acceptance**		
Enthusiast	Would recommend to others	5
Intermediary		4
Satisfied	Would do it again	3
Intermediary		2
Accepts	Would repeat it without hesitation	1
Denies	Would not repeat it	0

**Table 3:** English translation of the Romanian-translated MSTS Score for the lower limb.

*Description*	*Data*	*Score*
**Pain**		
No pain	No medication	5
Intermediary		4
Moderate/No invalidity	Non-narcotic analgesia	3
Intermediary		2
Moderate/Intermittent invalidity	Intermittent narcotics	1
Severe/Continuous invalidity	Continuous narcotics	0
**Function**		
No restriction	No invalidity	5
Intermediary		4
Recreational restriction	Minor invalidity	3
Intermediary		2
Partial occupational invalidity	Major invalidity	1
Total occupational invalidity	Complete invalidity	0
**Emotional acceptance**		
Enthusiast	Would recommend to others	5
Intermediary		4
Satisfied	Would do it again	3
Intermediary		2
Accepts	Would repeat it without hesitation	1
Denies	Would not repeat it	0
**Walking support**		
None		5
Intermediary		4
Support		3
Intermediary		2
1 crutch		1
2 crutches		0
**Walking**		
Unlimited		5
Intermediary		4
Limited		3
Intermediary		2
Online inside		1
Not independently		0
**Gait**		
Normal		5
Intermediary		4
Minor cosmetical defect		3
Intermediary		2
Major cosmetical defect		1
Major invalidity		0

**Table 4:** Patients’ complications classified by the Henderson classification after endoprosthetic reconstruction [26].

*Category*	*Complications after Endoprosthetic Reconstruction* *(n = Patients, %)*	*Complications after Bio Logical Reconst Ruction* *(n = Patients, %)*
*Mechanical*	I A: - I B: -	I A: - I B: 2, 11.1%
	II A: - II B: 2, 7.1%	II A: - II B: 8, 44.4%
	III A: 8, 28.6% III B: 4, 14.3%	III A: - III B: 6, 33.3%
*Non-Mechanical*	IV A: 2, 7.1% IV B: 2, 7.1%	IV A: - IV B: 2, 11.1%
	V A: - V B: -	V A: - V B: -
*Pediatric*	VI A: - VI B: -	VI A: 4, 22.2% VI B: -

**Table 5:** MSTS score for the upper limb in the study population for the first test.

MSTS Item	Mean	Standard Deviation	Range
*Pain*	4.87	0.33	0–5
*Functional activities*	3.66	0.90	0–5
*Emotional acceptance*	4.12	0.98	0–5
*Hand positioning/use of external supports*	3.54	1.12	0–5
*Manual dexterity/walking ability*	3.79	0.92	0–5
*Lifting abilities/gait*	3.33	0.85	0–5

**Table 6:** MSTS score for the upper limb in the study population for the retest.

MSTS Item	Mean	Standard Deviation	Range
*Pain*	4.85	0.33	0–5
*Functional activities*	3.75	0.83	0–5
*Emotional acceptance*	4.25	0.78	0–5
*Hand positioning/use of external supports*	3.66	0.99	0–5
*Manual dexterity/walking ability*	3.87	0.78	0–5
*Lifting abilities/gait*	3.5	0.58	0–5

## Data Availability

On request from the corresponding author, the data are not publicly available due to privacy and ethical reasons.
